# PCR-Based *Legionella* Risk Evaluation of Drinking Water Systems—An Empirical Field Evaluation

**DOI:** 10.3390/microorganisms13061311

**Published:** 2025-06-04

**Authors:** Markus Petzold, Nicole Zacharias, Sarah Uhle, Laurine Kieper, Nico Tom Mutters, Thomas Kistemann, Christiane Schreiber

**Affiliations:** 1 Institute of Medical Microbiology and Virology, University Hospital Dresden, Dresden University of Technology, Fetscherstraße 74, 01307 Dresden, Germany; 2 Institute for Hygiene and Public Health, University Hospital Bonn, Venusberg-Campus 1, 53127 Bonn, Germany; 3 Department of Geography, University of Bonn, Meckenheimer Allee 166, 53115 Bonn, Germany

**Keywords:** *Legionella*, qPCR, culture, monitoring, drinking water, hygiene

## Abstract

Pathogens in water systems pose potential health risks. Several countries provide guidelines and risk management strategies for clean water systems. Regarding legionellae, culture-based methods are still the gold standard, whereas molecular methods such as quantitative real-time PCR (qPCR) are controversially discussed among experts as an alternative. It remains questionable as to whether monitoring by qPCR contributes to sustainable water hygiene and effective health prevention. Drinking water samples from 101 buildings were culture-based analyzed to determine the legionellae concentration, along with qPCR tests. The negative predictive values for *Legionella* spp. and *L. pneumophila* qPCR regarding the cultivation method were 100% and 98%, respectively. As *Legionella* spp. DNA is ubiquitously detected, the positive predictive value was low. *L. pneumophila* DNA was in 18% of the drinking water samples detected by qPCR, among which only 7% was quantifiable. Neither gold-standard methods of cultivation nor qPCR methods alone are suitable to monitor the risk to health by legionellae in water environments adequately. To overcome methodical difficulties, the benefits of a strategic integration of qPCR alongside cultivation methods should be applied to develop a comprehensive protocol for the stepwise analysis of water samples, which can be implemented in international regulatory frameworks in the future.

## 1. Introduction

Identifying and assessing potential pathogen risks in water systems is a key focus of numerous national and international guidelines. These guidelines aim to prevent the contamination of drinking water by pathogens, emphasizing construction and operational factors that could promote the growth of opportunistic pathogens. Analyzing water samples for waterborne pathogens like *Legionella* spp., *Pseudomonas aeruginosa*, nontuberculous mycobacteria, and coliform bacteria is an important component of water monitoring [1].

The standard method for detecting and counting bacteria in water samples is cultivation. However, this approach has limitations, including poor recovery rates and long incubation periods. Furthermore, bacteria can transition to a viable but non-culturable (VBNC) state due to factors like nutrient depletion, metals, or UV light, leading to potential underestimation [2,3].

*Legionella* spp. are a prominent example of opportunistic pathogens in drinking water systems. These Gram-negative bacteria reside in biofilms, where they are ingested by protozoa like amoebae, replicate within the host cells, and are released to infect other hosts or settle in biofilms [4,5]. Aerosol-producing water systems, such as spas, cooling towers, and building plumbing, are high-risk, as *Legionella*-containing aerosols can cause the severe pneumonia known as Legionnaires’ disease (LD) [6,7]. Incidences of LD have increased globally in recent decades, leading many countries to implement monitoring policies for legionellae in plumbing systems and cooling towers [8,9,10]. 

Fundamental aspects of legionellae-monitoring policies include alert or action levels based on colony-forming units, which can trigger interventions like flushing, disinfection, or prohibiting water usage. In Germany, *legionella* monitoring in hot-water systems (PWH) involves sampling of buildings with a water heater with a volume > 400 L and/or >3 L volume in the pipes from heater to the outlet which is the furthest away. Buildings that provide water to the public (e.g., schools and hospitals) have to be monitored annually. Private buildings need to be monitored every three years, provided that the previous figure was under action levels. In addition, as soon as LD is reported, the public health authorities can sample potential sources. Samples are taken at water heaters, recirculation pipes, and representative peripheral taps, with an action level of 100 *Legionella* spp. per 100 mL implemented since 2003 [11].

The standard culture-based protocol for detecting and counting *Legionella* bacteria in water samples is described in ISO 11731 [12]. In Germany, this standard is supplemented by detailed guidelines from the German Environment Agency, which are part of generally accepted codes of practice updated over time [13]. These regulations define specific action levels for *Legionella* spp. detection, such as ≥100 CFU 100 mL^−1^ (e.g., requiring resampling in the whole building, and a risk assessment followed by technical or organizational improvement), >1000 CFU 100 mL^−1^ (e.g., immediate measures to reduce the concentration, resampling, and risk assessment followed by improvement), and >10,000 CFU 100 mL^−1^ (e.g., immediate disinfection and/or usage restrictions, risk assessment, and renovation of the drinking water plumbing system (DWPS)).

Recent studies in Germany found *Legionella* spp. contamination in 13–31% of samples from large private buildings, with 11–20% exceeding the 100 CFU 100 mL^−1^ action level [14,15,16]. Drinking water plumbing systems provide a suitable habitat for *Legionella*, so monitoring these bacteria is important for public health [17]. In an international context, studies showed that legionellae occurrence ranged from an average of 12.5% in cold drinking water (PWC) to 54.0% in PWH in areas with untreated groundwater [18]. Thus, for health prevention reasons, the surveillance and monitoring of legionellae in drinking water plumbing system are necessary.

In addition to cultivation, various methods have been described for the detection and quantification of legionellae in water samples [19]. During the last decade, qPCR methods became a widely accepted alternative tool for the fast and reliable detection of legionellae in clinical and environmental samples. This method is able to overcome the limitations of cultivation by detecting even VBNC cells. Nevertheless, the detection of DNA in water samples is not free of flaws, as it also detects free DNA and, if applied, the detection of VBNC requires a special sample pre-treatment. In particular, the detection of free DNA in samples leads to a considerable overestimation of the presence of the actual living—and therefore potentially infectious—bacteria, including accompanying pathogenic risks [20]. Often, scientists and authorities attempt to compare qPCR results with culture-based results directly—still a virtually impossible task [21,22].

When discussing the detection of legionellae, several considerations are relevant, including sensitivity, selectivity, and specificity. For example, the optimal cultivation method for detecting *Legionella* spp. other than *L. pneumophila* [23], the VBNC state of bacteria, the difficulty in distinguishing between dead and living cells, and the exclusivity of qPCR systems to detect *Legionella* spp. are all important factors.

Several studies have recommended a qPCR-based screening strategy for detecting legionellae in water samples as part of risk management or surveillance efforts. One significant advantage of qPCR over cultivation is its speed, with results available in several hours compared to 10 days with cultivation. This is particularly important when processing large numbers of samples or when rapid results are needed, such as in outbreak scenarios [24,25].

However, in Germany, the use of qPCR as a method for analyzing water samples is subject to certain conditions. According to current legislation, qPCR is not yet recognized as a standard method for evaluating water quality and safety, except in outbreak situations. Other studies with similar approaches focussed on other aspects, such as municipal water distribution systems, public buildings (schools, offices), hotels, cooling towers, or swimming pools, were carried out in countries with the general use of biocides in DWPSs [25,26,27]. This study collected samples and analyzed representative data from DWPSs across Germany only from multi-apartment buildings using both culture and qPCR methods in parallel.

## 2. Material and Methods

### 2.1. Sampling Procedure

**Figure 1:**
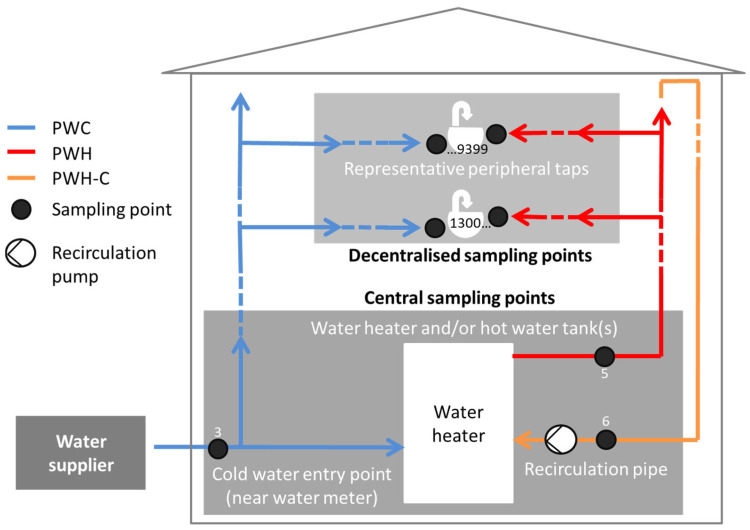
Schematic representation of sampling points within a building. Legend: (3), incoming cold water (PWC); (5), hot water (PWH) after the heater/tank; (6), recirculating PWH; and representative peripheral sampling points with numbers from 1300 to 9399.

In total, water samples from 101 premise plumbing systems, mainly multi-family houses (>80%) located in different areas of Germany with central heaters (volume of >400 litres) and recirculation lines were sampled between 2016 and 2020 in accordance with an extensive standardized protocol. This includes PWC samples right after the water metre (Figure 1), indicated as sampling point ‘3’, PWH samples after the water heater/hot water tanks, indicated as sampling point ‘5’, and the recirculating pipe right before re-entering the tank, indicated as sampling point ‘6’. Furthermore, PWC and PWH samples were taken at representative peripheral taps that have sampling point numbers ranging from ‘1300’ to potentially ‘9399’. The nomenclature for those peripheral sampling points is a 4-digit number (AABB), where AA corresponds to the database number of the riser and BB is the number of a peripheral sampling point of that riser. Samples were taken according to ISO 19458:2006 [28]. The samples from sampling points ‘3’, ‘5’, and ‘6’ are all sampled after 1 L flush (purpose b). In the periphery (point of use), the 1st litre was taken as the water was consumed (purpose c), directly without any disinfection or flushing. After that, the removal of aerators and either thermal (flaming) or chemical disinfection (70% 2-propanol *v*/*v*) were applied prior to the sampling of the 2nd litre (purpose b). In addition, for peripheral samples, the 5th litre was also collected (litres 3 and 4 were discarded) and analyzed, which should be close to temperature constancy if the system is properly technically dimensioned. The modified sample volume was one litre collected in two 500 mL bottles, in order to gain sufficient material for comparative sample analysis in parallel. The samples were equally analyzed by three laboratories with 35 buildings (Lab 1), 32 buildings (Lab 2) and 34 buildings (Lab 3), respectively.

### 2.2. Legionellae Detection Using Culture Methods

All samples were cultivated on GVPC agar for the presence of *Legionella* spp. [12]. Both sample bottles from a sampling point were shaken and volumes were pooled for subsequent analyses. Water was assayed in parallel by filtering 100 mL and directly plating 1 mL equally on two plates (0.5 mL each). Colonies that grew on BCYE agar containing *L*-cystein and not on agar without *L*-cysteine were regarded as *Legionella* spp. We additionally confirmed each morphological variant (e.g., colour, size, and UV-light-positive) by using MALDI-TOF (Bruker, Bremen, Germany). *L. pneumophila* isolates were further typed by using a ‘Dresden panel’ [29]. The results of direct plating and filtration assays were counted separately and reported as extrapolated colonies per 100 mL of sample volume each. As specified by the German Federal Environment Agency, for disease-prevention reasons the assay with the highest (extrapolated) *Legionella* count per 100 mL, instead of an arithmetical mean, was stated as the final result [13].

### 2.3. Legionellae Detection Using Molecular Methods

For comparative molecular analyses, another 500 mL of subsamples was used. The presence of genetic material of *L. pneumophila* and *Legionella* spp. in the samples’ DNA extract was examined using certified and validated qPCR assays according to ISO/TS 12869 [30]. The three laboratories involved in this study were equipped with different systems: GeneDisc^®^ *Legionella* Duo on a GeneDisc Cycler (Lab 3) (PALL, Bruz, France), or iQ-check^®^ Quanti Kits separately for *Legionella* spp. and *L. pneumophila* (Lab 1 and Lab 2) (Biorad, Munich, Germany) on either a Roche lightcycler 480-II (Roche, Freiburg, Germany) (Lab 1) or a Biorad CFX96 (Lab 2). Preceding DNA extraction was carried out by using a Gene Extract DNA Extractor (Biorad, Munich, Germany) with the GeneDisk^®^ system (PALL, Bruz, France), or using the Aquadien Bacterial DNA Extraction and Purification Kit (Biorad, Munich Germany) in combination with iQ Check^®^ Quanti kits. The lower limit of detection (LoD) differed slightly from 80 genomic units (GUs) per 500 mL sample (iQ-Check^®^ kits) to 190 GU 500 mL^−1^ (GeneDisc^®^ Duo). The limit of quantification (LoQ) was determined being 608 GU 500 mL^−1^ (iQ-Check^®^ kits) and 950 GU 500 mL^−1^ (GeneDisc^®^ Duo). In order to visualize the different methods in tables and figures the LoD was numerically set at 10 GU 500 mL^−1^ and the LoQ was set at 200 GU 500 mL^−1^.

Internal proficiency testing between the participating laboratories ensured the comparability of results, although different assays and cyclers were used. Test samples containing *L. pneumophila* were prepared by the Lower Saxony public health authority (laboratory for proficiency testing according to ISO/IEC 17043:2010), which also acts as the official German coordinator for cultural *Legionella* spp. ring trials (for details about the proficiency test, see Supplementary Materials, Table S1).

Commercial qPCR systems designed for detecting Legionella spp. in water systems are typically validated according to the ISO 12869:2019 standard [30]. During validation, 16 non-*Legionella* species are tested as an exclusion panel to ensure that the PCR system does not recognize them as *Legionella* spp. However, water systems are complex and not fully understood in terms of microbial composition and interactions. Legionellae account for only about 0.5% of the drinking water microbiome [31], and other, unculturable bacteria species may carry similar DNA sequences to legionellae, leading to false-positive results.

To address these concerns, we conducted an extensive examination of 16 drinking water subsamples from different apartments across Germany using DNA- and culture-based methodologies (Table S1). Our goal was to verify the specificity of the molecular tests. We analyzed the subsamples via cultivation according to ISO 11731 and used MALDI-TOF to identify putative legionellae colonies.

In a substudy, we verified the above-mentioned commercial kits with another (off-label use) commercial kit and, additionally, four published PCR protocols from the literature. A commercial kit from EuroClone (DUPLICα, Siziano, Italy) was developed for the detection of *Legionella* spp. in respiratory samples. DNA was extracted using a Gene Extract DNA Extractor (PALL). Processing of the qPCR was performed in accordance with the manufacturers’ protocol. Target gene sequences and primers of all kits were unknown to the authors, despite requesting information from the manufacturers. In addition, four well-described PCR-based methods obtained from the scientific literature with known gene sequences and primers targeting *Legionella* spp. were used for comparison in accordance with the published protocols: The method described by Miyamoto et al. [32] comprises two consecutive PCR runs (nested PCR; nPCR) and targets the 16S rDNA. Ratcliff et al. [33] developed a method that amplifies the conserved *mip* gene (macrophage infectivity potentiator). The melting-curve-based PCR system described by Benitez and Winchell [34] targets the tmRNA *ssrA* gene, and finally a PCR system for the 16S rDNA gene developed by Lesnik et al. [35] was used (Table S1). Since for these methods the target sequences are known, we generated the sequences of the amplified PCR products, if possible, by using Sanger sequencing (Institute of Molecular Cell Biology and Genomics, Dresden) and performed a nucleotide search by using BLAST v2.15.0 [36].

## 3. Results

### 3.1. Cultural Detection of Legionellae

This study investigated the presence of legionellae in 101 plumbing systems using two methods: culturing of *Legionella* spp. and molecular detection of *Legionella* spp. as well as *L. pneumophila* genetic material in parallel. Samples were collected from both public water heater (PWH) and public water cooler (PWC) plumbing systems.

The direct plating method detected *Legionella* spp. in 34 out of 865 samples (3.74%), while the filtration method detected legionellae in 77 out of 866 samples (8.47%). The lower number of samples analyzed using the direct plating method was due to the extensive growth of interfering colonies, which were excluded from the analysis.

In terms of the plumbing systems in buildings, 25% of the buildings tested positive for legionellae in at least one sample. Furthermore, 24 samples (2.3%) exceeded the German technical action level of 100 CFU 100 mL^−1^ (Table 1, Table S1).

Notably, the detection rate for legionellae was higher in the periphery (point of use) compared to the samples taken at the PWC entrance or the PWH samples around the heater (Table 1).

### 3.2. Molecular Detection of Legionellae

Of the 866 samples analyzed, 838 (96%) were tested for the presence of *L. pneumophila* genes using qPCR assays. The majority of these samples (688; 82%) had concentrations of *L. pneumophila* DNA below the LoD (<80–190 GU 500 mL^−1^). However, in 150 samples (18%) *L. pneumophila* DNA was detected. Among these, 63 (7.5%) results were above the LoQ (>608–950 GU 500 mL^−1^) and were quantifiable.

**Figure 2:**
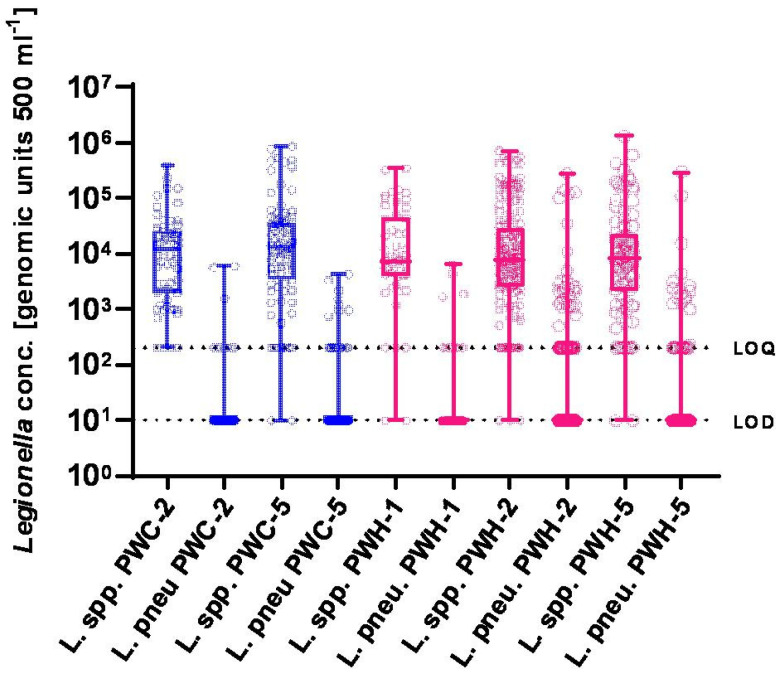
Distribution of *Legionella* concentration (GU/500 mL^−1^) in water samples. Samples were distinguished by sampling point (PWC: cold water, PWH: hot water, and number 2 or 5 indicted the sampled litre). In the figure, single data are shown as circles. For the statistical analysis and visualization, data below the LOD were labelled as 10, and values > LOD but <LOQ were labelled as 200 to allow an ordinal scale. Where evaluable, median values are marked as horizontal bars within the boxes showing 25^th^ percentile to 75^th^ percentile. Vertical bars indicate the whole range between minimum and maximum concentrations for each sample category; there are no outliers in the dataset. GU: genomic unit; LOQ: limit of quantification; and LOD: limit of detection.

The quantifiable results showed *L. pneumophila* DNA concentrations ranging from 2.3 × 10^2^ to 2.9 × 10^5^ GU/500 mL, with a median of 2.1 × 10^3^ GU/500 mL. Half of the samples fell within the range of 1.3 to 4.3 × 10^3^ GU/500 mL (Table 2, Figure 2). In addition, 494 samples were analyzed for the presence of *Legionella* spp. DNA by qPCR. Only 9 samples (1.8%) had concentrations below the LoD. Another 49 samples showed low concentrations of *Legionella* spp. DNA (>LoD but <LoQ). The remaining 436 samples (88.2%) were positive and quantifiable for *Legionella* spp., with concentrations ranging from 4.5 × 10^2^ to 1.3 × 10^6^ GU 500 mL^−1^, and a median of 1.2 × 10^4^ GU 500 mL^−1^.

As some qPCR runs were inhibited, we were unable to include all samples for the analysis.

### 3.3. Comparability of Results by Culture vs. DNA Detection

Given the lack of a quantitative correlation between qPCR and culturing methods [21,22,37], we chose to compare the qualitative classification of qPCR results (positive/negative) with those obtained by culturing (Table 3). We were able to obtain cultural and molecular results for 838 (*L. pneumophila*) and 494 (*L*. spp.) samples, respectively. We calculated the positive predictive value (PPV), negative predictive value (NPV), sensitivity, and specificity using fourfold tables (culture versus qPCR) with the following categories: correct-positive (a), correct-negative (b), false-positive (c), and false-negative (d). Our analysis revealed a positive predictive value (PPV) of 11.43% for *Legionella* spp. and 21.19% for *L. pneumophila* for the qPCR method. This means that only 11% and 21%, respectively, of the positive qPCR results were confirmed by *Legionella* spp. isolates obtained by culturing. Notably, almost all samples contained DNA fragments of legionellae (98.20%). The negative predictive value (NPV) indicates the probability that the culture method will be negative when the qPCR results are negative. We found an NPV of 97.99% for *L. pneumophila* and 100% for *Legionella* spp. (Table 4).

### 3.4. Comparison of Molecular Legionella Detection Methods

For *Legionella* DNA detection, we used three commercial kits (PALL GeneDisc, BioRad iQ-Check, and EuroClone DUPLICα) in parallel. While GeneDisc and iQ-Check were developed for detecting *Legionella* sp. and *L. pneumophila* in water samples, the EuroClone test was developed for detecting Legionella spp. in human respiratory samples. We also included four PCR-based methods from the literature for comparison (Table S1).

Our results showed that at least two methods confirmed the presence of legionellae in a sample. Notably, the nested PCR method of Miyamoto et al. [32] was positive for all samples and provided DNA sequences that matched with already-deposited Legionella sequences in the GenBank database, confirming the general presence of legionellae in all water samples.

The commercial tests predominantly yielded positive results, with the iQ-Check test for *Legionella* spp. showing 100% accordance with the nested PCR method. The EuroClone system, the method by Lesnik et al. [35], and the GeneDisc system performed similarly, while the melting-curve-based method detected legionellae in only six of sixteen samples. The *mip*-specific PCR was negative for all samples and not suitable for the direct detection of legionellae in water samples.

Regarding the performance of the tests on the *L. pneumophila* level, there were four samples from which sequences of *L. pneumophila* DNA were generated. These results were confirmed by the iQ-Check and GeneDisc tests, as well as the cultivation method.

## 4. Discussion

The present study confirms the widespread presence of *Legionella* spp. DNA in plumbing water systems in Germany, as almost all tested samples for *Legionella* spp. by qPCR were positive. This finding is in line with previous studies in Germany and Europe [14,24,38]. In contrast, the positivity rate of the samples was 7.9% for *Legionella* spp. using the ‘gold standard’ cultivation method, which is consistent with previous studies [39,40]. Importantly, our study confirms a negative predictive value (NPV) of 97.99% for *L. pneumophila* qPCR (*n* = 846) and 100% for *Legionella* spp. qPCR (*n* = 499), and demonstrates that negative cultural results can be predicted reliably by negative qPCR in multi-apartment buildings. Similarly to the mentioned studies, our project showed that each method for itself has its limitations in the enumeration of legionellae, leading to overestimation (e.g., *Legionella* spp. qPCR) or underestimation (*L. pneumophila* culture). The use of qPCR in this study allowed for the detection of *Legionella* spp. DNA in water samples, but the high positive detection rate was due to the presence of dead or VBNC cells. This suggests that qPCR systems without differentiation between living and dead cells may not be useful for assessing water systems [24,25,41]. This study highlights the importance of considering the presence of viable pathogenic cells when assessing the health risk associated with legionellae in water environments as proposed by other colleagues [42,43,44].

The high positive detection rate of the *Legionella* spp. qPCR systems, compared to the standard cultivation method, was already criticized in other studies [45,46]. Generally, the cultural evidence of legionellae has several limitations. For instance, the formula of the used agar in the past and the incubation temperature used for cultivation strongly selected for *L. pneumophila*, and thereby often neglected the presence of other *Legionella* species [47,48]. This changed with the introduction of the BCYEα+AB, leading to higher legionella counts and a higher detection rate of non-*L. pneumophila* [23]. Similarly, Sylvestre and colleagues calculated the ratios between qPCR results and cultivation and argued that “higher measurements with qPCR compared to cultivation likely arise from the detection of unculturable cells and extracellular DNA” and cultivation might underestimate risks due to methodological errors [49].

The variability of *Legionella* spp. concentrations at the periphery of plumbing water systems is likely due to various microbiological–ecological factors, such as the variable detachment of biofilm and the release of *Legionella* spp. from amoeba [3]. The study by Lautenschlager et al. [50] demonstrated a linear increase in intact cell concentrations within the first 12 h of stagnation, highlighting the significance of stagnation as a contributor to microbial regrowth. Furthermore, Völker et al. [51] observed significant fluctuations in *Legionella* spp. concentrations (KBE) at the periphery even within a single day, while Bédard et al. [52] demonstrated the effect of stagnation on lowering the temperature of domestic hot water systems and increasing cultivable cells.

The present study revealed that the positivity rate (qPCR and cultivation) in the first litre of a PWH was higher than the second and fifth litres of a PWH, especially for *L. pneumophila*. This observation can be explained by the construction of the water systems, characterized by central PWH circulation and taps interconnected via short end pipes, typically no more than 3 litres in volume. Stagnation in proximity to the tap results in a gradual decline in PWH temperatures, thereby creating more favourable growth conditions for legionellae compared to the continuously circulating hot water.

The relevance of the detection of non-*L. pneumophila* species in environmental samples is often discussed, suggesting those species may not be clinically relevant. This is because the vast majority of Legionnaires’ disease cases are reported by urinary antigen tests that are limited to only detect *L. pneumophila* serogroup (Sg) 1 antigens, indicating a limited consideration of the actual burden of non-*L. pneumophila* Sg1 infections [8,15,19]. Surveillance data from several studies report that a proportion of 10–15% of the LD cases were caused by non-*L. pneumophila* Sg1 strains [9,53]. With regard to these numbers, it is inevitable to screen water samples not only for the presence of *L. pneumophila* but for all *Legionella* spp. A qPCR-based approach or any other method that specifically detects living cells might become a new standard for the screening of water systems as a first step.

The present study also highlights the need for a more nuanced understanding of the relationship between *Legionella* spp. DNA and viable pathogenic cells. The use of viability qPCR (vPCR) may be a suitable method for closing putative cultural detection gaps and normalizing the genetic load to the actually viable fraction of the bacteria [2,54,55,56]. Viability PCR typically uses chemicals such as propidium monoazide (PMA) or ethidium monoazide (EMA) that penetrate dead cells and bind to DNA, preventing PCR amplification. This allows researchers to selectively amplify DNA from live cells, while ignoring DNA from dead cells. This method also needs some improvement to remove all dead cells [57]. Further research is needed to explore the potential of vPCR and other molecular biological methods for assessing the health risk associated with legionellae in water environments [49]. Those efforts were recently described for cooling towers and might be addressed to drinking water as well [43].

The debate surrounding the critical steps and differences in parameters between culture-based and molecular-based *Legionella* monitoring remains ongoing. Cultivation methods can only detect viable legionellae that proliferate and are capable of forming colonies, whereas qPCR methods reveal that legionellae are ubiquitous in aqueous systems. The negative predictive value (NPV) of qPCR for both *Legionella* spp. and *L. pneumophila* suggests that qPCR could be used as a screening tool to identify samples requiring further cultivation and to exclude water samples that are unlikely to be potential sources of infection. Notably, we detected *L. pneumophila* DNA by qPCR in only 18% of the drinking water samples. Therefore, positive results should be promptly confirmed using culture-based methods to inform evidence-based decisions regarding culture-based action levels and to mitigate acute human health risks.

This study suggests that a validated and officially accepted decision tree could be a useful instrument in the future for assessing water systems. The initial question for those that create such a tree should be the aim of the testing. As described in this and others studies, the detection of legionellae in water systems varies depending on several factors, such as the time point of sampling, method of sampling and detection, which buildings and what species should be tested, and what thresholds will be implemented at what point [24,27,39,44,49,58]. These factors should be cautiously verified. Thus, a decision tree could take into account the results of qPCR and other molecular biological methods, as well as the presence of viable pathogenic cells. Additionally, the targeting of virulence factors might be helpful in drawing a clearer picture of water systems and revising as well as renewing public-health-related risk assessment in routine monitoring. The questions are which litre should be tested and which method will be used to detect legionellae in a water system.

## 5. Conclusions

Since 2003, Germany has implemented a technical action level for culture-based *Legionella* spp. concentration of 100 CFU 100 mL^−1^. In 2020 the European Union followed suit, adopting a similar culture-based approach with a concentration threshold of 1000 CFU L^−1^. The EU Drinking Water Directive also incorporates risk management, allowing for the use of additional analytical methods. To ensure clean water systems, maintenance, technical monitoring, and risk assessment strategies, such as water safety plans, often involve routine monitoring regimes.

The challenges posed by raw water sources and diverse microbial communities within pipe systems make it difficult to establish a common threshold or alert/action level. However, the current state of research suggests that neither conventional cultivation methods nor qPCR methods alone are sufficient for monitoring the health risks associated with legionellae in water environments. To address this, we advocate for the usage of alternative molecular methods, alongside the cultivation method, for the detection of living *Legionella* spp. cells in drinking water. Continuous monitoring is essential, and the more accurate it reflects the actual situation, the better a potential health risk can be assessed.

## Figures and Tables

**Table 1:** Overall samples obtained from different sampling points and the positivity rate for *Legionella* spp. using culture methods (DP: direct plating).

Sampling Point	Analyzed Samples—DP (% *L*. spp. Positive)	Analyzed Samples—Filter (% *L*. spp. Positive)
PWC (before heater)	96 (3)	96 (2)
PWH 2nd L (after heater)	85 (1)	85 (4)
PWH 2nd L (recirculation)	79 (1)	79 (4)
PWH 1st L (periphery)	76 (6)	76 (9)
PWH 2nd L (periphery)	229 (12)	229 (26)
PWH 5th L (periphery)	171 (6)	171 (16)
PWC 5th L (periphery)	172 (5)	173 (17)
Total	908 (34)	909 (77)

**Table 2:** Molecular detection of *Legionella* spp. and *L. pneumophila* using qPCR. Results are classified as <limit of detection (LoD), <limit of quantification (LoQ), and >LoQ.

Sampling Points	qPCR [<LoD]		qPCR [<LoQ]		qPCR [>LoQ]		
	*L.* spp. [%]	*L. pneumophila* [%]	*L.* spp. [%]	*L. pneumophila* [%]	*L.* spp. [%]	*L. pneumophila* [%]	Total *L.* spp./*L. p.*
PWC (before heater)	0 [0.00]	85 [88.54]	4 [6.56]	7 [7.29]	51 [92.72]	3 [3.16]	55/95
PWH 2nd L (after heater)	0 [0.00]	70 [82.35]	6 [12.77]	7 [8.24]	41 [87.23]	8 [9.41]	47/85
PWH 2nd L (recirculation)	0 [0.00]	64 [81.01]	7 [15.91]	9 [11.39]	37 [84.09]	6 [7.59]	44/79
PWH 1st L (periphery)	2 [3.92]	48 [81.35]	3 [5.88]	7 [11.86]	46 [90.19]	4 [6.78]	51/59
PWH 2nd L (periphery)	3 [2.97]	138[77.09]	11 [10.89]	24 [13.41]	87 [86.14]	17 [9.49]	101/179
PWH 5th L (periphery)	2 [2.08]	134 [79.29]	9 [9.38]	18 [10.65]	85 [88.51]	17 [9.49]	96/169
PWC 5th L (periphery)	2 [2.00]	148 [86.05]	9 [9.09]	16 [9.30]	88 [88.89]	8 [4.65]	99/172
Total	9 [1.82]	688 [82.10]	49 [9.91]	88 [10.50]	436 [88.26]	63 [7.52]	494/838

**Table 3:** Comparison of *Legionella* spp. and *L. pneumophila* results obtained by qPCR and culture (combined *L.* spp. and *L. pneumophila*). Only results that are above the limit of detection and are found to be positive by culture and/or qPCR were included. Samplings that were negative for both were excluded.

	**qPCR**	**Culture Positive [%]**		**Culture Negative [%]**		**Total**
*Legionella pneumophila*	Positive	a	32 [3.81]	c	119 [14.18]	150
	Negative *	b	14 [1.67]	d	681 [81.17]	688
*Legionella* spp.	Positive	a	56 [11.42]	c	434 [88.57]	490
	Negative *	b	0 [0]	d	4 [0.82]	4

* below limit of detection. (a) correct-positive, (b) correct-negative, (c) false-positive, and (d) false-negative

**Table 4:** Summary of the positive and negative predictive values (PPV and NPV) for qPCR results compared to culture. The 95% confidence intervals are shown brackets.

	PPV (95% CI)	NPV (95% CI)
*Legionella pneumophila*	21.19 (17.27–25.72)	97.99 (96.91–98.69)
*Legionella* spp.	11.43 (11.29–11.57)	100 (93.62–100.00)

## Data Availability

The original contributions presented in this study are included in the article/Supplementary Materials. Further inquiries can be directed to the corresponding author.

## Supplementary Materials

The following supporting information can be downloaded at: https://www.mdpi.com/article/10.3390/microorganisms13061311/s1, Table S1: Data of sampling points. The table shows the data for eachbuilding and contains information about the sampling point, temperature and the detected legionellae (culture and/or qPCR).
